# Multimodal sensing and machine learning for continuous monitoring of agitation in dementia

**DOI:** 10.1002/alz70857_101188

**Published:** 2025-12-24

**Authors:** Marta Bono, Zhen Liu, Nick Van Helleputte, Maarten De Vos, Maarten Van Den Bossche

**Affiliations:** ^1^ KU Leuven, Leuven, Belgium; ^2^ imec, Heverlee, Belgium

## Abstract

**Background:**

With the increase of the average life expectancy, the number of people with dementia (PwD) is expected to increase to 152 million by 2050. Agitation is a very frequent and clinically important neuropsychiatric symptom of dementia, that is associated with more rapid progression of the disease, increased morbidity and mortality, and higher economical costs. However, there is currently no way to robustly detect agitation and there is a clear need to understand what causes agitation in each person with dementia. Current methods with questionnaires are very subjective, prone to bias and very intermittent. This is an important factor in the current sub‐optimal prevention and treatment of agitation, and the overuse of potentially dangerous psychotropic medication in PwD.

**Method:**

We designed a unique platform combining non‐obtrusive physiological sensors worn by the PwD, location and environmental sensors, and ecological momentary assessment by staff, in order to continuously monitor agitation, discover digital biomarkers of agitation and identify triggers for agitation both on an individual and population level.

**Result:**

So far, we included 42 patients in our study, that were monitored for at least 7 consecutive days and nights, to form the largest database of its kind in the world. We will present our current findings on potential digital biomarkers of agitation in dementia. Data will also be presented on which environmental triggers are involved in different types of agitation. Finally, we will elaborate on lessons learned during this project that can guide future research in this field.

**Conclusion:**

Multimodal sensing combined with machine learning techniques, could allow for more continuous, objective and quantifiable monitoring of agitation in dementia. This could lead to a better evaluation of interventions, lower need for potentially dangerous psychotropic drug use, less caregiver stress and lower economic costs. Our results support the initial hypothesis of population heterogeneity and they represent the base for personalized agitation detection and treatment evaluation using physiological markers and environmental triggers.

